# Editorial: Molecular studies and therapeutic approaches on BRCA-associated cancers

**DOI:** 10.3389/fmolb.2026.1821951

**Published:** 2026-03-26

**Authors:** Audesh Bhat, Xiaoqiang Wang, Preyesh Stephen

**Affiliations:** 1 Department of Life Science, Central University of Karnataka, Kalaburgi, India; 2 Department of Cancer Biology and Molecular Medicine, City of Hope, Duarte, CA, United States; 3 CHU de Québec Research Center and Department of Molecular Medicine, Laval University, Québec, QC, Canada

**Keywords:** BRCA-associated cancers, companion diagnostic (CDx), FDA approval, homologous recombination deficiency (HRD) score, PARP inhibitors (PARPi)

As researchers continue to achieve important breakthroughs in cancer research, one of the most critical areas of focus remains the understanding and treatment of cancers associated with *BRCA1/2* mutations. Both *BRCA1* and *BRCA2* genes are key players in maintaining genomic stability (Roy et al., 2011). When mutated, cells lose the ability to repair DNA damage, leading to the accumulation of mutations and an increased risk of developing cancers, particularly those of the breast and ovaries (Kuchenbaecker et al., 2017). Studies have unraveled a complex network of molecular pathways disrupted in BRCA-deficient cells, importantly cell cycle regulation and apoptotic pathways (Yoshida and Miki, 2004; Quaas et al., 2026). The *BRCA1/2* mutations, though relatively rare in the general population, account for a significant proportion of breast, ovarian, and other cancers, and their implications for both patients and healthcare systems are profound (Kuchenbaecker et al., 2017). The past decade has seen remarkable progress in therapies specifically targeting BRCA-mutated tumors, most notably PARP inhibitors (PARPi), although resistance remains a growing concern (Turk and Wisinski, 2018; Shao et al., 2021). BRCA-mutated tumors also exhibit higher levels of genomic instability, generating tumor-specific neoantigens that enhance immune visibility and spurring interest in combining immune checkpoint inhibitors with DNA-targeted therapies such as PARPi. Another promising therapeutic frontier is synthetic lethality, targeting genes or pathways that, when inhibited in conjunction with *BRCA* mutations, lead to cancer cell death (Lord and Ashworth, 2017). In this Research Topic, we bring together the latest molecular studies and therapeutic approaches to BRCA-associated cancers, shedding light on emerging insights and potential avenues for future treatments.

A major contribution is the technical validation of an in-house homologous recombination deficiency (HRD) test for high-grade ovarian cancer developed by Nogueira Rodrigues et al. The GS Focus HRD assay, based on next-generation sequencing, demonstrated substantial concordance with the clinical gold standard MyChoice® HRD Plus CDx (κ = 0.8), achieving 90% accuracy, sensitivity, and specificity, with all *BRCA*1/2 mutations detected by the reference assay also identified by the new panel. Given the high prevalence of HRD in high-grade serous ovarian cancer and its central relevance for PARPi therapy, this validated assay represents a significant step toward improving global accessibility of reliable HRD diagnostics. Sun et al. used a bidirectional two-sample Mendelian randomization approach to investigate the causal relationship between breast cancer and thyroid neoplasms. The analysis identified a significant positive causal effect of breast cancer on malignant thyroid tumors (OR 1.291, 95% CI 1.143–1.458, *P* = 3.90 × 10^−5^), which remained significant after multivariable adjustment for smoking, alcohol, and BMI, suggesting that thyroid cancer screening may be warranted in breast cancer patients.

Two case reports expand the phenotypic spectrum traditionally associated with hereditary breast and ovarian cancer. Liu et al. report a 74-year-old male presenting with concurrent breast cancer and high-grade metastatic prostate cancer (Gleason score 8, T4N1M1) who carried a germline *BRCA2* mutation (c.5987dupC; p. R1997Kfs*6). After mastectomy and tamoxifen, the patient developed progressive prostate cancer with bone metastases; multimodal neoadjuvant therapy proved insufficient, and he underwent robotic-assisted radical prostatectomy. Germline testing of his children revealed previously unreported *BRCA2* variants, underscoring the importance of cascade genetic testing across generations. Griesler et al. describe a 47-year-old male with advanced non-seminomatous germ cell tumor carrying a pathogenic *BRCA1* germline mutation who developed unusually severe and prolonged pancytopenia during first-line chemotherapy, culminating in neutropenic sepsis. The authors discuss how *BRCA1* haploinsufficiency may impair bone marrow recovery, drawing parallels to hematotoxicity patterns observed in *BRCA1*-mutated breast cancer patients, and raise the possibility of employing PARPi in the event of recurrence.

**FIGURE 1:**
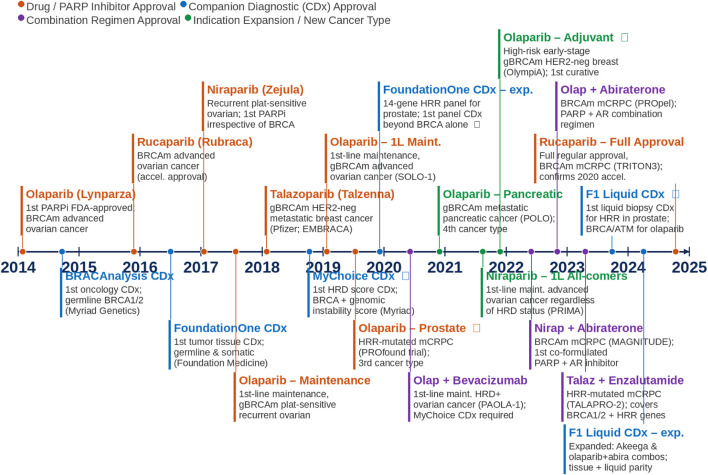
FDA approval timeline of PARP inhibitors and companion diagnostics in BRCA-mutated and HRD-positive cancers (2014–2025). Drug approvals (orange), companion diagnostics (blue), combination regimens (purple), and indication expansions (green) are shown by year.

Since the first FDA approval of a PARPi in 2014 for BRCA-mutated advanced ovarian cancer, the clinical application of *BRCA* and HRD testing has expanded considerably. Four PARPi (olaparib, rucaparib, niraparib, and talazoparib) have now been approved across ovarian, breast, prostate, and pancreatic cancers, with indications progressively moving from late-line salvage therapy toward first-line maintenance and adjuvant settings, reflecting growing confidence in the predictive value of *BRCA* and HRD biomarkers (Figure 1). The companion diagnostic (CDX) landscape has evolved in parallel: Myriad Genetics’ germline BRACAnalysis CDx (2014) covers germline *BRCA* identification; Foundation.

Medicine’ FoundationOne CDx (2016) extended testing to somatic tumor mutations; Myriad’s MyChoice CDx (2018) introduced an HRD genomic instability score enabling PARPi eligibility beyond *BRCA*-mutated patients; FoundationOne CDx was expanded to a 14-gene HRR panel for prostate cancer (2019); and liquid biopsy platforms have more recently extended testing to patients where tissues are unavailable (2023–2024). Olaparib (2022) adjuvant approval for high-risk early-stage *gBRCA*m HER2-negative breast cancer marked the first PARPi approval in a curative-intent setting, while combination PARPi plus androgen receptor pathway blockade established a new treatment approach for *BRCA*m metastatic castration-resistant prostate cancer. Together, these approvals reflect a broader shift: *BRCA* and HRD testing have become routine components of oncology workup across multiple tumor types.

All four articles in this Research Topic converge on the role of genes involved in homologous recombination (*BRCA1*, *BRCA2*, and related pathways) in disease risk, diagnostic classification, and therapeutic response. Drug resistance to PARPi remains a significant barrier requiring better combination strategies that target both tumors and resistance mechanisms. Not all *BRCA* mutations are alike, and identifying robust biomarkers to predict which patients will benefit most from specific therapies remains an important unmet need, as does developing effective treatments for metastatic BRCA-associated cancers. Together, these contributions enrich our understanding of cancer biology and highlight actionable paths toward better screening strategies, precision treatments, and equitable access to molecular testing worldwide.
